# The Ability of AhR Ligands to Attenuate Delayed Type Hypersensitivity Reaction Is Associated With Alterations in the Gut Microbiota

**DOI:** 10.3389/fimmu.2021.684727

**Published:** 2021-06-29

**Authors:** Osama A. Abdulla, Wurood Neamah, Muthanna Sultan, Hasan K. Alghetaa, Narendra Singh, Philip Brandon Busbee, Mitzi Nagarkatti, Prakash Nagarkatti

**Affiliations:** Department of Pathology, Microbiology, and Immunology, School of Medicine, University of South Carolina, Columbia, SC, United States

**Keywords:** aryl hydrocarbon receptor, forkhead box P3, interleukin 17, gut microbiome, short-chain fatty acids

## Abstract

Aryl hydrocarbon receptor (AhR) is a ligand-activated transcription factor that regulates T cell function. The aim of this study was to investigate the effects of AhR ligands, 2,3,7,8-Tetrachlorodibenzo-p-dioxin (TCDD), and 6-Formylindolo[3,2-b]carbazole (FICZ), on gut-associated microbiota and T cell responses during delayed-type hypersensitivity (DTH) reaction induced by methylated bovine serum albumin (mBSA) in a mouse model. Mice with DTH showed significant changes in gut microbiota including an increased abundance of *Bacteroidetes* and decreased *Firmicutes* at the phylum level. Also, there was a decrease in *Clostridium* cluster XIV and IV, which promote anti-inflammatory responses, and an increase in *Prevotella copri* that facilitates pro-inflammatory responses. Interestingly, treatment of mice with TCDD attenuated the DTH response, induced Tregs, suppressed Th17 cells in the mesenteric lymph nodes (MLNs), and reversed the gut microbiota composition toward normalcy. In contrast, FICZ exacerbated the DTH response, induced heightened Th17 cells, and failed to cause a major shift in gut microbiota. Furthermore, TCDD but not FICZ caused an increase in the levels of short-chain fatty acids (SCFA), n-butyric acid, and acetic acid. Administration of sodium butyrate into mice with DTH suppressed the response, increased Tregs, and reduced Th17 cells IL17. Butyrate also caused an increase in the abundance of *Clostridium* and a decrease in *Prevotella.* Lastly, TCDD, as well as butyrate but not FICZ, were able to inhibit proinflammatory Histone deacetylases (HDACs) class I and II. Together, our data suggest that AhR ligands, such as TCDD that suppress DTH response, may mediate this effect by reversing the gut dysbiosis induced during this inflammatory response, while FICZ may fail to suppress the DTH response because of its inability to overturn the dysbiosis.

## Introduction

Delayed-type hypersensitivity (DTH) is a type IV hypersensitivity response involved in a large number of clinical disorders, including contact dermatitis, drug-induced allergy, allogenic transplant rejection, and pathogenesis of certain autoimmune diseases (1). It also plays a major role in protection against intracellular bacterial infections, such as tuberculosis, while causing major tissue injury when not regulated (2, 3). DTH in the mouse has been used extensively to study the immune mechanisms and develop therapeutic drugs to treat DTH. One of the models involves the use of methylated bovine serum albumin (mBSA), which has been shown to activate primarily Th17 cells (4–6).

The aryl hydrocarbon receptor (AhR) is a vital immunomodulator and a ligand-dependent transcriptional factor that belongs to the basic helix-loop-helix (7). It is widely expressed in T cells, B cells, macrophages, NK cells, and dendritic cells (8). Activation of AhR can be induced by its exogenous and endogenous ligands (8). Depending on the nature of such ligands, the effect of AhR activation can be very diverse, from activation to suppression of immune response (9). For example, TCDD, an exogenous potent ligand, activates AhR leading to induction of Tregs and suppression of immune system (6, 10, 11), while FICZ, an endogenous ligand, also activates AhR but exacerbates inflammation by inducing Th17 cells (6, 10). Several dietary AhR ligands have also been shown to induce Tregs while suppressing Th17 cells (6, 12).

Recent studies have shown that AhR plays a crucial role in regulating intestinal homeostasis through epithelial cell renewal from intestinal stem cells (ISCs). Additionally, AhR regulates the maintenance of a homeostatic symbiosis between gut microbiome and the host that could lead to protection from pathogenic intestinal infection or chronic inflammation (13, 14). The gut microbiota is a diverse and complex community of gut microorganisms, including bacteria, viruses, archaea, fungi, and protozoans (15). The gut microbiota has a mutual relationship with its host and is engaged in the maintenance of the immune system, metabolic process, and protection against pathogens, in healthy individuals (16). Gut microbiota provides the host multiple advantages by regulating the host immunity (17, 18).

Tregs and Th17 cells are two essential lymphocyte subsets with disparate functions (19). Tregs/Th17 imbalance has often been found to be an important factor for metabolic- or immunologic disorder-associated diseases, including numerous types of allergic diseases (20, 21), chronic inflammations (22, 23), autoimmune diseases (24), and cancers (25, 26). Several studies have shown that intestinal microbiota is closely associated with the balance of Th17 and Tregs (27). Certain segmented filamentous bacterium (SFB) were shown to induce CD4+ T helper cells that produce IL-17 and IL-22 in the lamina propria (27). Furthermore, SFB was able to induce autoimmune arthritis through Th17 cells (28). In contrast, certain *Clostridia* promoted colonic Treg accumulation that maintained the immune hemostasis (29). Recently, it has been observed that gut microbiota-derived metabolites, short-chain fatty acids (SCFAs), stimulate the differentiation and development of Th17 cells and Tregs in humans (30). Sodium butyrate (BUT) treatment increased the number of Tregs but decreased the number of Th17 cells in experimental autoimmune uveitis through Nrf2/HO-1 pathway (31). Thus, understanding the relationship between intestinal microbiota and the equilibrium of Tregs/Th17 cells balance would be crucial to the cure of many diseases, triggered by an imbalance of these two T-cell subsets.

In the current study, we investigated the effect of two diverse AhR ligands (TCDD and FICZ) on the gut microbiome that led to Tregs/Th17 imbalance in a DTH mouse model. The results demonstrated that AhR ligands TCDD and FICZ have opposite effects with TCDD causing attenuation of DTH, inducing Tregs in the MLNS, altering the gut microbiota that promote anti-inflammatory activity, and induing butyric acid which facilitates Treg induction. In contrast, FICZ had opposite effects at all these levels. The current study demonstrates that while DTH response promotes proinflammatory gut microbiota and Th17 response in the MLNs, AhR ligands, such as TCDD, which reverse the dysbiosis, attenuate the DTH response, whereas FICZ, which fails to impact the dysbiosis, fails to suppress the DTH response. These data suggest that the ability of AhR to suppress DTH may be associated with gut dysbiosis.

## Materials and Methods

### Animals

Female C57BL/6 (BL6) mice, 8 to 10 weeks old with an average weight of 20 g, were purchased from Jackson Laboratories (Bar Harbor, ME). The mice were housed at the University of South Carolina School of Medicine Animal Resource Facility, in pathogen-free conditions and light- and temperature-controlled facility that allowed ad libitum access to water and diet. All animal experiments performed in this study were approved by the University of South Carolina Institutional Animal Care and Use Committee animal protocol (IACUC).

### Induction of Delayed-Type Hypersensitivity and Treatment With AhR Ligands, and Sodium Butyrate

To generate DTH in mice, mBSA was used as an antigen as described previously (32, 33). Briefly, C57BL/6 mice were randomized and sensitized with subcutaneous injection (100 µl/hind flank) of 1.5 mg/ml of mBSA (Sigma-Aldrich, MO) emulsified in complete Freund’s adjuvant (CFA, Sigma Aldrich). Six days later, mice were re-challenged by intradermal injection of 10 mg/ml mBSA in PBS (20 μl) into the footpads. All mice were then separated into four groups: control (PBS), vehicle (corn oil), TCDD (10 µg/kg body weight), and FICZ (50 µg/kg body weight). These doses of TCDD and FICZ used in this study were based on the differential effects of these ligands on Tregs and Th17 cells as shown in our previous studies (34) and those from others (10, 35). On day 5, each group of mice was treated with vehicle, TCDD, or FICZ by intraperitoneal (i.p.) route as described previously (34, 36). The footpad thickness of the mice was measured using an engineer’s calipers 48 h after the secondary challenge on day 8. The footpad swelling response was calculated in percentage using the following formula: [(thickness (mBSA rechallenged footpad) − the thickness (PBS rechallenged footpad))/thickness (PBS rechallenged footpad)]/100. For butyrate administration experiments, after the induction of DTH as described above, mice were divided to four groups: control (naïve), sodium butyrate (BUT), mBSA+Veh, and mBSA+BUT. On day 5, BUT dissolved in water (500 mg/kg body weight) was administered orally, one dose a day for 3 days.

### Antibodies, Reagents, and Chemicals

TCDD was kindly provided by Dr. Steve Safe (Institute of Biosciences & Technology, Texas A&M Health Sciences Center, College Station, Texas). FICZ was purchased from Enzo Life Sciences (Farmingdale, NY). Both TCDD and FICZ were dissolved in DMSO and diluted in corn oil for use. Sodium butyrate, corn oil, and mBSA were purchased from Sigma-Aldrich (St. Louis, MO). RPMI 1640, l-glutamine, penicillin-streptomycin, HEPES, PBS, and FBS were purchased from Invitrogen Life Technologies (Carlsbad, CA). Fluorophore labeled monoclonal antibodies (mAbs), such as BV785-conjugated anti–CD45, FITC–conjugated anti-CD3, APC/cy7-conjugated anti-CD4, and Alexa Fluor 700-conjugated anti-CD8 used for the flow cytometry, were purchased from Bio Legend (San Diego, CA) and Thermo Fisher (Grand Island, NY). For FoxP3 staining, we used FoxP3 Transcription Factor Buffer Set, and for IL-17 we used intracellular (IC) Fixation and Permeabilization Buffer from Thermo Fisher (Grand Island, NY).

### Cellular Phenotyping by Flow Cytometry

To evaluate the effect of AhR ligands on immune cells in mice with DTH, mesenteric lymph nodes (MLNs) were harvested 48 h after the secondary challenge and treatments. Briefly, single-cell suspensions of MLNs were prepared and then cultured for 24 h in the presence of the Leukocyte Activation Cocktail (BD Biosciences, San Diego, CA) for 5 h to activate the cells. The cells were collected, washed, and stained using anti-mouse fluorophore-labeled mAbs. The cells (1 × 10^6^ cells) were incubated together with Fc Blocker purchased from BD Biosciences (San Diego) for 10 min and then treated with conjugated anti-mouse monoclonal antibodies (BV785-conjugated anti–CD45, FITC–conjugated anti-CD3, APC/cy7-conjugated anti-CD4, and Alexa Fluor 700-conjugated anti-CD8) from Biolegend (San Diego, CA) for 30 min at 4°C. The cells were washed twice using FACS buffer (1× PBS containing 2% fetal bovine serum). The stained cells were analyzed using a flow cytometer (BD FACSCelesta), and data were analyzed using FlowJo v10 software. Foxp3/transcription factor staining buffer set and IC fixation and permeabilization (Thermo Fisher, Waltham, MA) were used for staining cells for Foxp3 and IL17 following the manufacturer’s instructions. Briefly, the cells were first stained for cell surface marker (CD4), and after washing, the cells were fixed using fixation/permeabilization buffer. The fixed cells were washed with PBS and then stained using anti-mouse antibodies (APC-conjugated anti-Foxp3, FITC-conjugated anti–IL-17) purchased from BD Biosciences (San Diego). The stained cells were analyzed by a flow cytometer (BD FACSCelesta) and data were analyzed using FlowJo v10 software.

### Behavioral Pain Assays of Mice With DTH

To test the pain-associated DTH response, mice with DTH and treated with vehicle, TCDD, or FICZ were subjected to standard von Frey filament test and hot-plate test. The von Frey filament test was performed as described previously (37) Briefly, each mouse was transferred to a plastic cage fitted with a metal mesh floor and von Frey filaments (0.008 to 1.4 g) (North Coast Medical, Inc, Morgan Hill, CA, USA) having the ability to exert a different force to the animal paw, from the weakest to the strongest, until the paw was withdrawn. Paw withdrawal was observed as a positive reaction, and mechanical sensitivity was characterized by withdrawal threshold (37). The hot plate test was used to assess the heat threshold (38). In the hot plate test, an unrestrained mouse was placed on a metal surface maintained at a constant temperature of 50°C (Bioseb, Pinellas Park, FL, USA), and the response latency, which is the time taken to observe a nocifensive behavior, was recorded, which included hind paw withdrawal or licking and stamping (39).

### Real-Time Quantitative PCR to Determine the Gene Expression in Cells from MLNs

To determine the expression of FoxP3, TGF-β, IL-10, IL-17, and RORγT in MLNs, RT-qPCR was performed as described earlier (34). Briefly, total RNA isolated from the MLN cells was used to generate cDNA using cDNA synthesis kit from Qiagen (Germantown, MD, USA) and following the protocol of the company. SSO Advanced™ SYBR green PCR kit obtained from Bio-Rad (Hercules, CA, USA) was used to perform RT-qPCR on CFX96 RT-qPCR system (BioRad). The following primer sets (Primer Bank, Harvard Medical School) were used for RT-qPCR.

Foxp3 (F 5′CCCATCCCCAGGAGTCTTG3′; R 5′ACCATGACTAGGGGCACTGTA3′)
TGF-β (F 5′CGCTGCCCTTAAAAATATGGC3′; R 5′GAGCCCCCTTTGTCTGAACTG3′)
IL-10 (F 5′CCCATTCCTCGTCACGATCTC3′; R 5′TCAGACTGGTTTGGGATAGGTTT3′) IL-17 (F 5′CTCCTGCTTCTAGGCTGGTTG3′; R 5′CCACCTGGCACTTCGAGTTAG3′)
RORγT (F 5′CAAGTTTGGCCGAATGTCC3′; R 5′CTATAGATGCTGTCTCTGC3′)
IL-22 (F 5′GTCAACCGCACCTTTATGCT3′; R 5′ CATGTAGGGCTGGAACCTGT3′).

RT-qPCR was performed using the following PCR cycles (40 cycles) and under these conditions: initial activation step (15 min at 95°C), denaturing temperature (15 s at 94°C), annealing temperature (30 s at 60°C), and extension temperature and fluorescence data collection (30 s at 70°C) were used. Using NE ¼ 2_∆∆Ct, where Ct is the threshold cycle to detect fluorescence, normalized expression (NE) of mRNAs was calculated, and fold change of mRNA levels was normalized to β-actin (a housekeeping gene, *ACTB*).

### Assessment of Cytokines in Serum

To assess cytokines in serum, we performed an enzyme-linked immunosorbent assay (ELISA). In brief, blood was collected on day 8 (euthanizing day) and serum was separated and stored at −80°C until analysis. ELISA kits for pro-inflammatory (IL17A and IL6) and anti-inflammatory (IL-10) were obtained from BioLegend (BioLegend, San Diego, CA, USA). Following the protocol of the company, ELISA was performed using the serum. Absorbance was measured at 450 nm using a Victor2 1420 counter (Wallac) as described previously (33).

### Bacterial 16S rRNA Sequencing and PiCRUSt Functional Analysis

Colonic microbiota profiling was determined using 16S rRNA sequencing method and MySeq (Illumina, Illumina Way, San Diego, CA, USA) available in our laboratory, and phylogenetic analysis of bacterial colonization was determined by reconstruction of Unobserved States (PiCRUSt). 16S rRNA gut microbial profiling and SCFA quantification was done as previously described in our laboratory (40). Briefly, cecal flushes were collected from control, mBSA+Veh, mBSA+TCDD, and mBDS+FICZ groups after euthanasia and stored at −80°C until DNA isolation. For 16S rRNA sequencing, DNA was extracted from colonic flush contents by using the QIAamp DNA Stool Mini Kit (Qiagen, Valencia, CA, USA) according to instructions from the manufacturer. The 16S rRNA V3-V4 hypervariable regions of bacterial DNA libraries were prepared by amplification with added Illumina adapter overhang nucleotide sequences and sequencing with Illumina (San Diego, CA, USA) MiSeq platform. Nephele platform (https://nephele.niaid.nih.gov) from the National Institute of Allergy and Infectious Diseases (NIAID), Office of Cyber Infrastructure and Computational Biology (OCICB), Bethesda, MD was used for analyzing the sequenced reads (41).

To evaluate phylogenetic and possible metabolomic alterations within samples QIIME FASTQ paired-end with chimera removal, open reference, and SILVA rRNA database (Silva_99) options were used. For PiCRUSt data, a closed reference against the Greengenes database (Greengene_99) selection was used. Operational taxonomic unit (OTU) tables created from Nephele were additionally subjected to Linear Discrimination Analysis of effect size (LEfSe), which was provided by the Huttenhower group (https://huttenhower.sph.harvard.edu/galaxy/) (42).

RT-qPCR was performed to validate the presence of the following bacteria: *Clostridium* cluster XIV (coccoides group) and respective primer pairs (F 5′-AAATGACGGT ACCTGACTAA-3′; R 5′-CTTTGAGTTTCATTCTTGCGAA-3′); *Clostridium* cluster IV (leptum group) (F 5′-TTACTGGGTGTAAAGGG-3′; R 5′-TAGAGTGCTCTTGCGTA-3′); *Prevotella copri* (F 5′-CCGGACTCCTGCCCCTGCAA-3′; R 5′-GTTGCGCCAGGCACT GCGAT-3′). Using the Qiagen miScript kit (Qiagen, Valencia, CA, USA), cDNA synthesis was performed according to the protocol of the company. Using Bio-Rad SSO advanced SYBR Green PCR reagents, RT-qPCR was performed on a CFX96 RT-qPCR system (BioRad). Fold change of the bacteria was determined using the ∆∆CT method relative to a Eubacteria (Forward: 5′-ACTCCTACGGGAGGCAGCAGT-3′ Reverse: 5′-ATTACCGCGGCTGCTGGC-3′) as a control.

### SCFA Analysis From Cecal Flushes

Quantification of SCFAs of cecal flushes collected from experimental groups was performed as previously described (43). Briefly, cecal contents (100 mg) were suspended in deionized water and then acidified using 25% metaphosphoric acid. The contents were centrifuged at 12,000*g* for 15 min at 4°C and then filtered using Ultra-free MC columns obtained from Thermo Fisher Scientific. HP 5890 gas chromatograph configured with flame-ionization detectors (GC-FID) was performed for SCFA analysis and 0.1 mM 2-ethyl butyric acid was used as an internal standard (IS) for all samples and standards. Varian MS Workstation (version 6.9.2) software was used for quantification and to detect the concentration of acetic acid, propionic acid, n-butyric acid, isovaleric acid, and valeric acid in the samples.

### Microscopic Evaluation of the Colon Tissues

Colon tissues were collected from DTH mice on day 8. Colon tissues were fixed in 4% paraformaldehyde (PFA) overnight and then embedded in paraffin. Microtome sections (5 μm) were cut and transferred to slides. The colon tissues were stained with Hematoxylin and Eosin (H&E) to evaluate cellular morphology and infiltration into the colon. Histopathological scoring parameters were based on previously published reports (44). Images of stained sections were taken using a Keyence microscope (Itasca, IL, USA).

### Statistical Analysis

Statistical analyses were performed using GraphPad Prism software (GraphPad Software version 8). The data were presented as mean ± SEM in groups of five or more mice. The Student’s t-test was performed to compare data between two groups. A one-way ANOVA test was used to compare three or more groups followed by post hoc analysis using Tukey’s method. Statistically significant differences were illustrated in figures as *p<0.05, **p<0.01, ***p<0.001, ****p<0.0001.

## Results

### Effect of TCDD and FICZ on Delayed-Type Hypersensitivity Reaction

**Figure 1:**
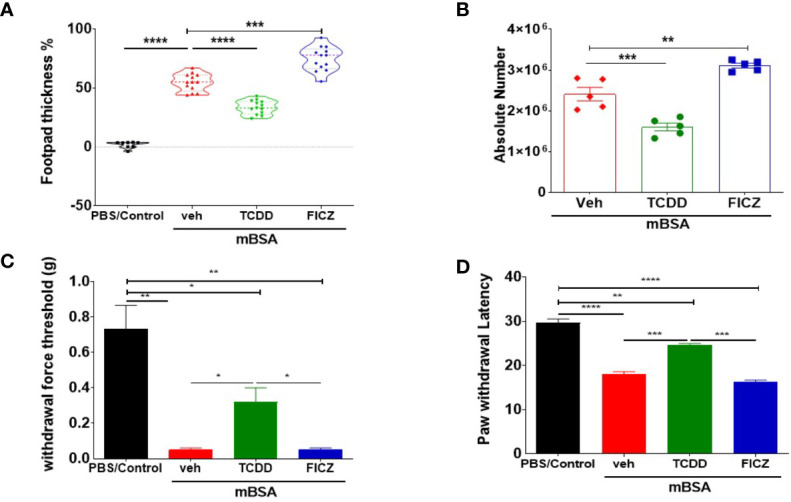
TCDD and FICZ exhibit opposite outcomes in mBSA-induced DTH in mice. DTH was induced by injecting mBSA followed by treatment with vehicle, TCDD, or FICZ. **(A)** Showing the percentage change in footpad thickness. **(B)** Absolute number of cells in mesenteric lymph nodes. **(C)** Von Frey filament test (0.008–1.4 g) was used to evaluate withdrawal force threshold of DTH reaction. **(D)** Latency for paw withdrawal on the hot plate test. The statistically significant differences between the groups were calculated using one-way ANOVA. Vertical Bars represent mean ± SEM and significant differences in total cell numbers are shown with asterisks (*p < 0.05, **p < 0.01, ***p < 0.001, ****p < 0.0001).

mBSA was used to generate DTH in C57BL/6 mice. There were four groups: PBS/control, mBSA+Veh, mBSA+TCDD, and mBSA+FICZ. The data obtained from this experiment showed that mBSA+Veh–treated mice showed a significant increase in footpad swelling, an indicator of DTH response, when compared with PBS/control (**Figure 1A**). TCDD treatment (mBSA+TCDD group) significantly reduced DTH responses by reducing the footpad thickness in comparison with mBSA+Veh group (**Figure 1A**). In contrast, FICZ treatment (mBSA+FICZ group) significantly increased the DTH responses by increasing the footpad thickness when compared with mBSA+Veh group (**Figure 1A**). Moreover, upon examination of the cellularity of mesenteric lymph node (MLN), there was also a significant increase in the total number of cells in the mBSA+Veh group when compared with PBS/control. Whereas mBSA+TCDD group showed a significant reduction in the total number of the cells in MLN when compared with mBSA+Veh group (**Figure 1B**), the mBSA+FICZ group showed a significant increase in the total number of the cells in MLN when compared with the mBSA+Veh group (**Figure 1B**).

To test if the mBSA-induced inflammation and development of DTH in mice were associated with pain, we performed behavioral pain assays. The mice from both mBSA+Veh and mBSA+FICZ groups demonstrated significantly reduced threshold force for hindlimb withdrawal response to von Frey filament filaments (**Figure 1C**) in comparison to PBS/control and mBSA+TCDD mice that showed increase in the threshold force. In addition, a hot plate test for pain measurement in mice (**Figure 1D**) showed that mice from mBSA+Veh and mBSA+FICZ had a significant reduction in paw withdrawal latency response, which is the time taken to see a nocifensive behavior (hind paw withdrawal or licking) to the hot plate, when compared with PBS/Con and mBSA+TCDD groups. Collectively, these data demonstrated that AhR ligands, TCDD, and FICZ, had opposite effects on mBSA-induced DTH response and associated pain in mice (**Figures 1A–C**).

### TCDD and FICZ Affect CD4+ T Cells But Not CD8+ T Cells in Mice With DTH

**Figure 2:**
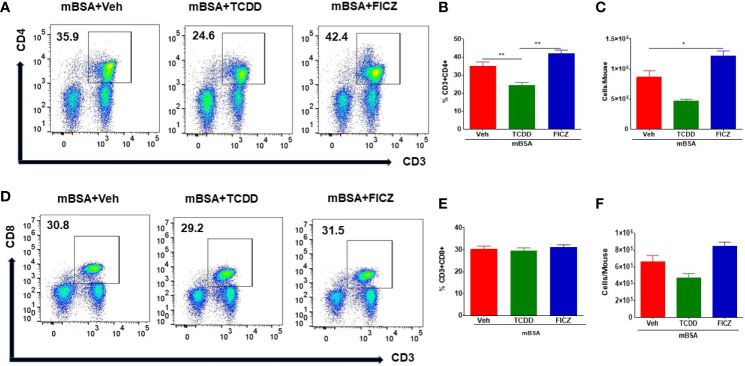
Effect of TCDD or FICZ on CD3+ and CD8+ T cells in the MLNs. DTH was induced by injecting mBSA followed by treatment with vehicle, TCDD, or FICZ. **(A, D)** Representative plots from FlowJo software analysis of flow cytometry data showing CD3+CD4+ and CD3+CD8+ cells in the MLNs. **(B, E)** Show percentages of CD3+CD4+ cells and CD+CD8+ T cells, respectively in groups of 5 mice. **(C, F)** Absolute cell numbers of CD3+CD4+ cells and CD+CD8+ T cells, respectively, in groups of 5 mice. The absolute numbers of T cell subsets were calculated based on the absolute number of cells found in the MLN multiplied by the percentage values of CD3+CD4+ cells or CD+CD8+ T cells obtained by flow cytometry, divided by 100. The statistically significant differences between the groups were calculated using one-way ANOVA. Vertical bars represent mean ± SEM and significant differences between groups were indicated by asterisks (*p < 0.05, **p < 0.01).

Because our goal was to test the effect of the AhR ligands on the gut-associated immune response, we tested the immunological changes occurring in the mesenteric lymph nodes (MLNs) of mice with DTH. **Figures 2A, D** show representative flow cytometric data of CD3+CD4+ and CD3+CD4+ T cells, respectively, and panels B and E show data from groups of five mice. Also, panels C and F show the total number of CD3+CD4+ or CD3+CD8+ T cells per mouse, which was calculated based on the percentage and absolute numbers of cells present in the MLNs. As shown in **Figures 2A–C**, there was a significant decrease in both the percentage and numbers of CD3+CD4+ T cells in the mBSA+TCDD group when compared to the mBSA+Veh group. On the other hand, there was a significant increase in the percentage and total number of CD3+CD4+ T cells in the mBSA+FICZ group when compared with mBSA+Veh (**Figures 2A–C**). However, neither mBSA+TCDD treatment nor mBSA+FICZ treatment had a significant effect on the total number of CD3+CD8+ cells when compared with the mBSA+Veh group (**Figures 2D–F**). Taken together, these data demonstrated that TCDD reduced the number of CD4+ T cells in the mesenteric lymph nodes, whereas FICZ increased the number of CD4+ T cells when compared with vehicle-treated mice with DTH, with no significant effect on CD8+ T cells.

### TCDD and FICZ Differentially Regulate the Generation of Tregs and Th17 Cells in Mice With DTH

**Figure 3:**
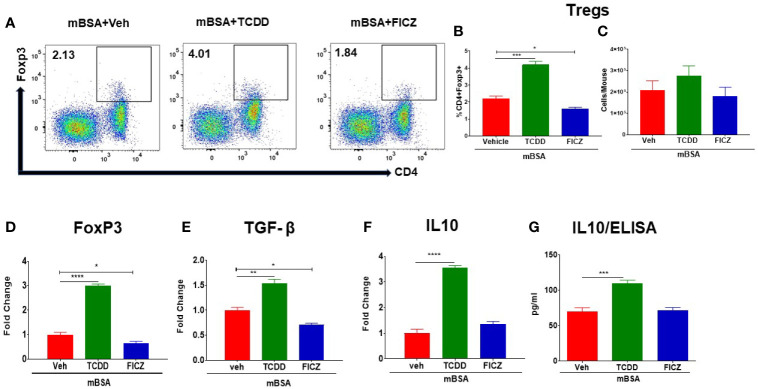
Effect of TCDD and FICZ on FoxP3+ Tregs during DTH response in the MLNs. DTH was induced by injecting mBSA followed by treatment with vehicle, TCDD, or FICZ. Cells harvested from the MLNs and analyzed. **(A)** Representative plots of flow cytometry data showing CD4+ Foxp3+ cells in MLNs. **(B)** Percentage of CD4+Foxp3+ cells from groups of 5 mice. **(C)** Total number of CD4+Foxp3+ cells from groups of 5 mice. qRT-PCR mRNA expression of Foxp3 **(D)**, TGF-β **(E)**, and IL-10 **(F)** in MLN cells. **(G)** Detection of IL10 by ELISA in the serum. The statistically significant differences between the groups were calculated using one-way ANOVA. Vertical bars represent mean ± -SEM and significant differences between groups are shown with asterisks (*p < 0.05, **p < 0.01, ***p < 0.001, ****p < 0.0001).

Next, we investigated the effect of TCDD and FICZ on Tregs and Th17 cells in the MLNs of mice with DTH. As shown in **Figures 3A, B**, flow cytometric data showed that TCDD treatment caused a significant increase in the percentage and but not the numbers of Tregs in MLNs of mice with DTH when compared with vehicle-treated mice (**Figures 3A–C**). In contrast, FICZ treatment caused a significant reduction in the percentage, but not the number, of Tregs when compared with the mBSA+Veh group (**Figures 3A–C**). Next, we evaluated the expression of Foxp3, a transcription factor for Tregs, as well as Treg effector molecules, such as TGF-β and IL10, by RT-PCR. As shown in **Figures 3D–F**, there was a significant increase in FoxP3 (**Figure 3D**), TGF-β (**Figure 3E**), and IL10 (**Figure 3F**) expression in the mBSA+TCDD group when compared with the mBSA+Veh group. Treatment with FICZ in contrast caused a significant decrease in Foxp3 and TGF-β expression with no significant changes in IL10 expression (**Figures 3D–F**). When the expression of IL10 was determined by ELISA in the serum, we noted a significant increase in IL10 in the mBSA+TCDD group when compared with mBSA+Veh mice, whereas FICZ did not cause significant change when compared with the vehicle control (**Figure 3G**). Taken together, these data demonstrated that TCDD increased the proportion of Tregs and induction of FoxP3, TGF-β, and IL-10 in the MLNs of DTH mice, whereas FICZ decreased the percentage of Tregs, FoxP3, and TGF-β in the MLNs of mBSA-induced DTH mice.

**Figure 4:**
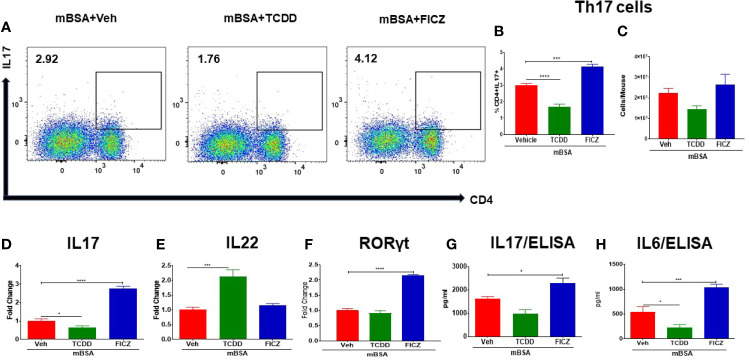
Effect of TCDD and FICZ on Th-17 cells in the MLNs of mice with DTH. DTH was induced by injecting mBSA followed by treatment with vehicle, TCDD or FICZ. Cells harvested from the MLNs and analyzed for Th-17 cells. **(A)** Representative plots from FlowJo software analysis of flow cytometry data showing CD4+IL7+ cells. Percentage **(B)** and total number **(C)** of CD4+IL17+ cells based on groups of 5 mice. **(D)** qRT-PCR based mRNA expression of IL17 **(D)**, IL-22 **(E)**, and RORγ **(F)** in MLN cells. Serum was collected on day 8 to determine levels of IL17 **(G)** and IL6 **(H)** by ELISA. Vertical bars represent mean ± SEM and significant differences between groups are shown with asterisks (*p < 0.05, ***p < 0.001, ****p < 0.0001) based on one-way ANOVA.

We also investigated the effect of TCDD and FICZ on the generation of Th17 cells in the MLNs of mice with DTH. There was a significant decrease in the percentage but not the numbers of Th17 cells in the MLNs of mBSA+TCDD mice when compared with mBSA+Veh mice (**Figures 4A–C**), In contrast, mBSA+FICZ mice showed a significant increase in the percentage but not the numbers of Th17 cells when compared with mBSA+Veh mice (**Figures 4A–C**). Upon evaluation of IL17 and RORγt expression (RT-qPCR) in MLN cells, there was a significant increase in both IL17 and RORγt expression in the mBSA+FICZ mice when compared with the mBSA+Veh mice (**Figures 4D, F** **)**. On the other hand, mBSA+TCDD group showed a significant decrease in IL17 expression when compared with mBSA+Veh mice (**Figure 4D**), whereas RORγt expression showed no significant change (**Figure 4F**). When IL-22 expression was assayed in the MLN cells, we noted that IL-22 expression was significantly increased in the mBSA+TCDD group when compared to the mBSA+Veh mice, while the mBSA+FICZ mice showed no significant change (**Figure 4E**). Furthermore, upon evaluation of IL17 and IL6 in sera by ELISA, there was a significant increase in both cytokines in the mBSA+FICZ group in comparison with mBSA+Veh mice (**Figures 4G, H** **)** while TCDD suppressed these cytokines (**Figures 4G, H** **)**. Taken together, these data demonstrated that AhR ligands, TCDD and FICZ, have opposite effects on Tregs and Th17 cells during mBSA-induced DTH reaction in mice.

### AhR Ligands, TCDD and FICZ, Alter the Gut Microbiota and SCFA in mBSA-Induced DTH Mice

**Figure 5:**
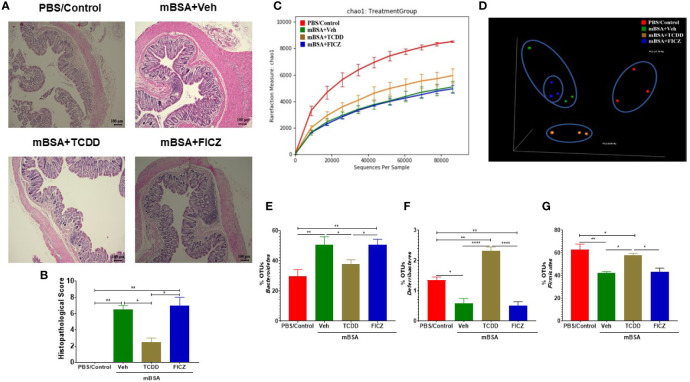
Effect of DTH response and treatment with TCDD and FICZ on the gut microbiome. DTH was induced by injecting mBSA followed by treatment with vehicle, TCDD or FICZ. Cecal flushes were collected for microbiome analysis. **(A)** H&E (10X magnification) staining of histological sections of the colon from all four groups of mice. **(B)** Bar graph showing histopathological scores of H&E-stained colons from mice (n = 3). **(C, D)** 16S rRNA sequencing from the cecal flushes was performed on PBS/Control (n = 3), mBSA+Veh (n = 3), mBSA+TCDD (n = 3), and mBSA+FICZ (n = 3) groups. Sequenced reads were analyzed using Nephele to generate chao1 alpha diversity **(C)** and beta diversity PCOA **(D)** plots. **(E–G)** Individual bar graphs are depicted for the following at the phylum level: Bacteroidetes **(E)**, Deferribacteres **(F)**, and Firmicutes **(G)**. Vertical bars represent mean ± SEM and significant differences between groups are shown with asterisks (*p < 0.05, **p < 0.01, ****p < 0.0001). based on one-way ANOVA.

Next, we investigated microbial dysbiosis in the gut of mice with DTH. We first evaluated the health of the colons of all the four groups (PBS/control, mBSA+Veh, mBSA+TCDD, and mBSA+FICZ) histologically. Analysis of histological data of the colons showed that there was a significant cell infiltration in the mBSA+Veh group when compared with the PBS/control group (**Figure 5A**). However, the mBSA+TCDD group showed a reduction in the number of infiltrating cells in the colon in comparison with the mBSA+Veh group (**Figure 5A**). mBSA+FICZ group, on the other hand, exhibited a slight increase in the number of infiltrating cells in the colon when compared with the mBSA+Veh group (**Figure 5A**). Histological scores of colons showed that mBSA+TCDD group showed a significant decrease in scores, whereas mBSA+FICZ group showed similar scores when compared with the mBSA+Veh group (**Figure 5B**). These data suggested that mBSA was able to induce inflammation in the colon of DTH mice, and that although TCDD caused an attenuation, FICZ failed to decrease the inflammatory response in the colon.

**Figure 6:**
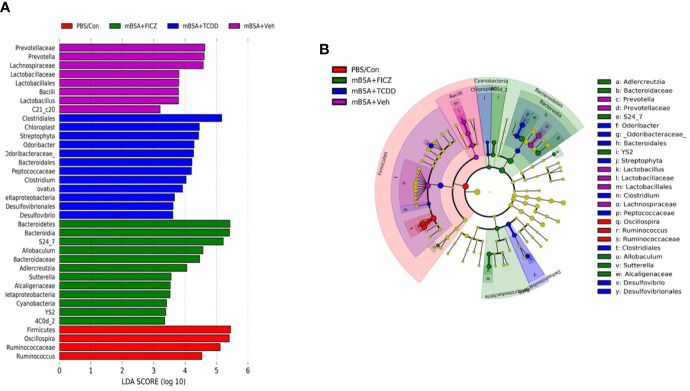
LefSE analysis of unique microbiota found in different treatment groups of mice with DTH. 16S rRNA sequencing from the cecal flushes was performed on PBS/Control (n = 3), mBSA+Veh (n = 3), mBSA+TCDD (n = 3), and mBSA+FICZ (n = 3) experimental mice and OTU data were subjected to LefSE analysis to generate cladogram **(A)** and LDA scores **(B)** among all four groups. For LefSe data, the alpha factorial Kruskal-Wallis test among classes was set to 0.05, and the threshold on the logarithmic LDA score for discriminative features was set at 3.

**Figure 7:**
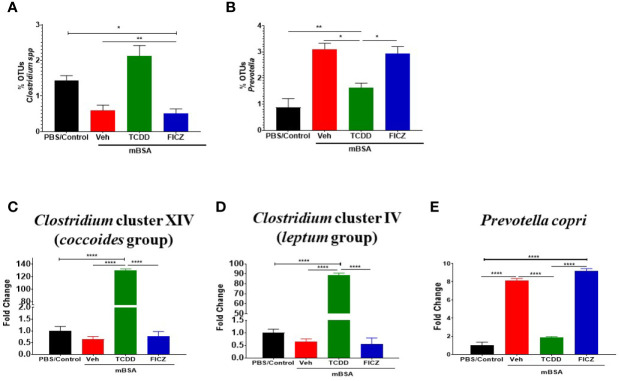
TCDD and FICZ alter the microbiome and microbiome-associated metabolome in mice with DTH response. **(A)** Bar graph depicting the percent OTUs of *Clostridium* generated from 16S rRNA sequencing **(B)** Bar graph depicting the percent OTUs of *Prevotella* generated from 16S rRNA sequencing **(C)** PCR validation and fold change of *Clostridium* cluster XIV (*coccoides* groups) in PBS/Control (n = 3), mBSA+Veh (n = 3), mBSA+TCDD (n = 3), and mBSA+FICZ (n = 3). **(D)** PCR validation and fold change of *Clostridium* cluster IV (*leptum* groups). **(E)** Bar graph depicting PCR validation and fold change of *Prevotella copri*. Vertical bars represent mean ± SEM and significant differences between groups are shown with asterisks (*p < 0.05, **p < 0.01, ****p < 0.0001) based on one-way ANOVA.

Next, we determined the gut microbiome in mice with DTH and treated with vehicle or TCDD or FICZ. To this end, we performed bacterial 16S rRNA gene sequencing using Illumina MiSeq platform and primers targeting the V3 and V4 variable regions. Sequences were analyzed using 16S QIIME paired-end pipeline available online by the National Institute of Health (NIH). We used the Nephele platform (release 1.6 which uses QIIME 1.9.1) for analysis of MySeq data to characterize differences in gut microbiome diversity, phylogeny, and function (45). Upon assessment using Chao1 rare fraction measure, we noted that DTH induction using mBSA had lowered the abundance of bacteria in the gut when compared to mice that did not receive mBSA (**Figure 5C**). Also, vehicle and FICZ treatments of DTH mice showed similar abundances of bacteria in the gut, whereas TCDD treatment increased the abundances of bacteria in the gut when compared with these two groups (**Figure 5C**). Beta diversity analysis that measures the similarity or dissimilarity between various groups showed that all groups were well separated. However, mBSA+Veh and mBSA+FICZ bacteria were more closely clustered together, whereas the other two groups, PBS/control and mBSA+TCDD, showed clear separation from each other (**Figure 5D**). This suggested that the microbiome composition in the mBSA+TCDD was distinct when compared with mBSA+Veh. In contrast, mBSA+FICZ mice showed a close resemblance in microbiome composition with mBSA+Veh. At the phylum level, there were notable differences. Mice with DTH (mBSA+Veh) had an increased abundance of Bacteriodetes when compared with mice that did not receive mBSA (PBS/Control) (**Figure 5E**). mBSA+TCDD group showed a decreased abundance of Bacteriodetes when compared with the mBSA+Veh group, whereas the mBSA+FICZ group showed levels similar to that shown by mBSA+Veh (**Figure 5E**). Furthermore, mBSA+Veh and mBSA+FICZ groups had significantly lower levels of Deferribacteres and Firmicutes when compared with PBS/control (**Figures 5F, G**). In contrast, the mBSA+TCDD group showed an increased abundance of Deferribacteres and Firmicutes when compared with the mBSA+Veh and mBSA+FICZ groups (**Figures 5F, G**). To distinguish significantly altered bacteria among all the experimental groups, Linear discriminant analysis effect size (LefSe) (46) analysis (**Figure 6A**) and the corresponding cladogram (**Figure 6B**) were generated on the operational taxonomic unit (OTUs) from phylum to species level. Data showed that there were several bacteria found to be distinctly expressed in each of the four groups (**Figure 6A**). For example, *Clostridium* was shown to be significantly decreased in the bacteria abundance OTUs following DTH (mBSA+Veh) when compared to mice without DTH (PBS/Control) group, whereas in the mBSA+TCDD group, it was significantly increased while in the mBSA+FICZ group, it was similar to mBSA+Veh group (**Figure 7A**). In contrast, for *Prevotella,* DTH induction (mBSA+Veh) caused increased abundance when compared to mice without DTH (PBS/Control) (**Figure 7B**). In contrast, mBSA+TCDD–treated mice showed a decrease in *Prevotella* while the mBSA+FICZ group showed no significant change when compared to the mBSA+Veh group (**Figure 7B**).

To confirm the results obtained from sequencing post-Nephele and LefSe analyses, PCR was performed using primers specific for the following species: *Clostridium* cluster XIV (*coccoides *group) (**Figure 7C**), *Clostridium* cluster IV (*leptum* group) (**Figure 7D**), and *Prevotella copri* (**Figure 7E**). These results confirmed the data obtained from the sequencing. Specifically, *Clostridium* was found to be significantly increased in mBSA+TCDD mice when compared to other groups while *Prevotella copri* was found to be significantly decreased in mBSA+TCDD in comparison with mBSA+Veh and mBSA+FICZ (**Figures 7C–E**). Taken together, the phylogenetic data and PCR data supported that DTH induction leads to significant alterations in the colonic microbiota, and furthermore AhR ligands, such as TCDD, cause additional alterations. Interestingly, treatment of DTH mice with FICZ did not cause major alterations and behaved similar to the mBSA+Veh group.

**Figure 8:**
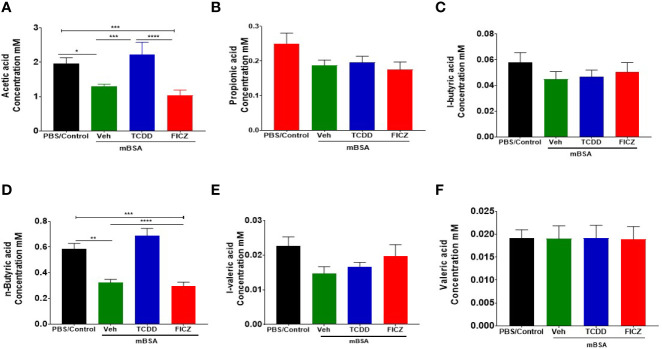
Concentrations of SCFAs from cecal flushes of DTH mice treated with TCDD or FICZ: DTH was induced by injecting mBSA followed by treatment with vehicle, TCDD or FICZ. Cecal flushes were analyzed as follows. **(A–F)** Concentrations of SCFAs from cecal flushes of PBS/Control (n = 5), mBSA+Veh (n = 5), mBSA+TCDD (n = 5), and mBSA+FICZ (n = 5). Vertical bars represent mean ± SEM and significant differences between groups are shown with asterisks (*p < 0.05, **p < 0.01, ***p < 0.001, ****p < 0.0001) based on one-way ANOVA.

In addition to microbial profiling, bacterial metabolomic SCFA production was also evaluated in the cecal flush samples collected from all four groups (**Figures 8A–F**). Data showed that two of the SCFA (acetic acid and n-butyric acid) were found to be significantly reduced in the mBSA+Veh and mBSA+FICZ group when compared to PBS/Control group, whereas the mBSA+TCDD group showed restoration to normal levels seen in PBS/Control group (**Figures 8A, D** **)**. Taken together, these results showed that AhR ligands behave differently with respect to SCFA induction during the DTH response, and that although TCDD treatment was able to restore some of the SCFA to normal levels, FICZ was unable to restore.

### Sodium Butyrate Inhibits HDACs and Ameliorates mBSA-Induced DTH in Mice

**Figure 9:**
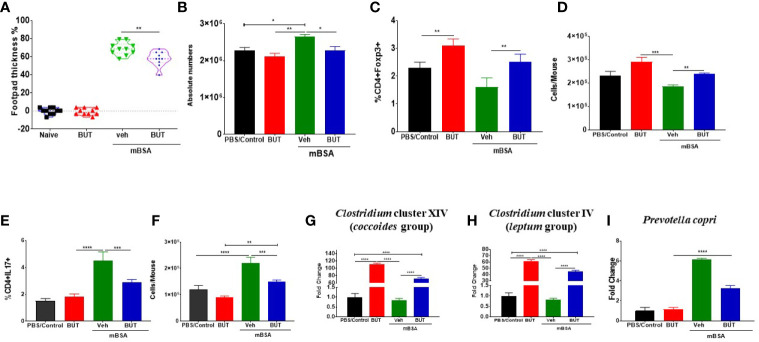
Effect of sodium butyrate (BUT) on DTH response, immune profile, and gut microbiota. DTH was induced as described in Figure 1 legend. Experimental groups consisted of: Naïve (n= 5), BUT (n = 5), mBSA+Veh (n = 5), and mBSA+BUT (n = 5). **(A)** Showing the percentage change in footpad thickness. **(B)** Absolute cell number in the mesenteric lymph nodes. Percentage **(C)** and total number **(D)** of CD4+Foxp3+ cells in mesenteric lymph nodes of various groups. Percentage **(E)** and total number **(F)** of CD4+IL17+ cells in the MLNs of various groups. Bar graph depicting PCR validation and fold change of *Clostridium* cluster XIV (*coccoides* groups) **(G)**, *Clostridium* cluster IV (*leptum* groups) **(H)**, and *Prevotella copri* **(I)** in cecal flushes of various groups. The statistically significant differences between the groups were calculated using one-way ANOVA. Vertical bars represent mean ± SEM and significant differences between groups are shown with asterisks (*p < 0.05, **p < 0.01, ***p < 0.001, ****p < 0.0001) based on one-way ANOVA.

Because TCDD induced higher levels of butyrate, we next tested the effect of oral administration of Sodium Butyrate (BUT) on DTH in mice. For this experiment, we took four groups of mice: naïve, mice with BUT (BUT), DTH mice with vehicle (mBSA+Veh), and DTH mice with BUT (mBSA+BUT). mBSA+BUT mice showed a significant reduction in the footpad thickness when compared with the mBSA+Veh group (**Figure 9A**). Moreover, upon examination of the cellularity of mesenteric lymph node (MLN), there was also a significant reduction in the total number of cells in the mBSA+BUT mice when compared with mBSA+Veh mice (**Figure 9B**). We also noted significant increase in the percentage and numbers of Tregs (**Figures 9C, D** **)** and a decrease in the percentage and numbers of Th17 cells (**Figures 9E, F** **)** in MLNs of the mBSA+BUT group when compared with the mBSA+Veh group.

Next, we investigated the effect of BUT treatment on the bacteria that were identified as significantly altered by AhR ligands. To that end, we performed RT-qPCR to determine the presence of *Clostridium* cluster XIV (*coccoides* group) (**Figure 9G**), *Clostridium* cluster IV (*leptum* group) (**Figure 9H**), and *Prevotella copri* (**Figure 9I**) in mice. The results demonstrated that treatment with BUT (mBSA+BUT) caused a significant increase in both *Clostridium* clusters when compared to the mBSA+Veh group (**Figures 9G, H**). Interestingly, BUT alone in naïve mice caused a significant increase in *Clostridium* clusters (**Figures 9G, H**). Treatment with BUT during DTH caused a significant decrease in *Prevotella copri* when compared with the mBSA+Veh group (**Figure 9I**). These data suggested that butyrate can suppress DTH, increase Tregs, and suppress Th17 cells as well as alter the levels of *Clostridium* and *Prevotella*, similar to TCDD.

### Effect of AhR Activation on HDAC During the DTH Response

**Figure 10:**
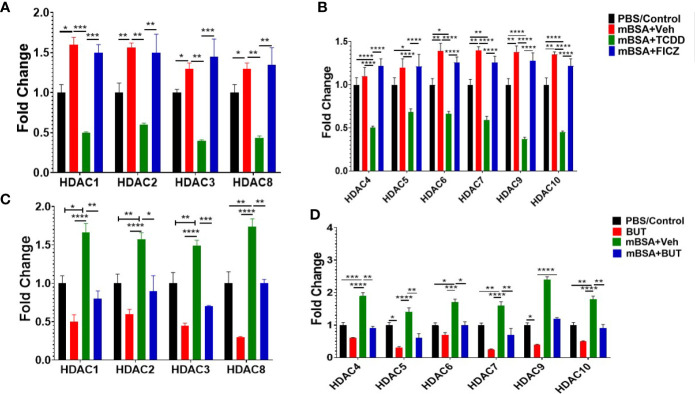
Treatment of mice bearing DTH with TCDD, FICZ or BUT leads to inhibition of HDAC. Expression of HDAC I **(A)** and HDAC II **(B)** was evaluated from MLNs isolated from experimental groups (PBS/Con, mBSA+Veh, mBSA+TCDD, mBSA+FICZ). PCR-based fold change in the expression of HDAC I **(C)** and HDAC II **(D)** was evaluated in MLN cells isolated from various groups. Vertical bars represent mean ± SEM and significant differences between groups are shown with asterisks (*p < 0.05, **p < 0.01, ***p < 0.001, ****p < 0.0001) based on one-way ANOVA.

Previous studies have shown that butyrate also inhibits HDAC activities which in turn exert anti-inflammatory activities (47). To that end, we also investigated HDAC expression in mice with DTH and treated with AhR ligands (TCDD and FICZ) or BUT. The results obtained from this experiment showed that TCDD (mBSA+TCDD) caused a significant decrease in HDAC I (HDAC 1, 2, 3, 8) expression in mice with DTH when compared mBSA+Veh group, and FICZ-treated (mBSA+FICZ) mice (**Figure 10A**). Also, mBSA+TCDD mice showed a significant decrease in HDAC II (HDAC 4, 5, 6, 7, 9, 10) expression when compared with mBSA+Veh mice and mBSA+FICZ mice (**Figure 10B**). Interestingly, administration of BUT (mBSA+BUT) also caused a significant decrease in HDAC I and HDAC II expression when compared with the mBSA+Veh group (**Figures 10C, D** **)**.

## Discussion

In the current study, we demonstrate that induction of DTH can alter the gut microbiota and that AhR activation during this process can have a significant impact on this dysbiosis with potential consequences on T cell responses and inflammation associated with the DTH response. Our study demonstrated that AhR ligands, such as TCDD and FICZ, have opposite effects on DTH response mediated by mBSA, with the former suppressing DTH while the latter, enhancing this response, consistent with our recent studies (34). Interestingly, in our previous report (34) on mBSA-induced DTH response, we focused on T-cell responses in the draining popliteal lymph nodes while in the current study, because we were interested in studying gut-associated microbiota and inflammation, resorted to MLNs. Thus, we were surprised to note that the changes brought about by AhR ligands in the MLNs were similar to that seen in the draining lymph nodes (34) in that TCDD caused induction of Tregs while suppressing Th17 cells, whereas FICZ had the opposite effects on these T cells. Furthermore, there was a significant increase in Foxp3, TGF-β, and IL10 expression in MLN as well a significant increase in IL10 concentration in the serum of mBSA+TCDD mice when compared with the mBSA+Veh group. FICZ, on other hand, caused a significant increase in the expression of IL17 and RORγ in MLN (**Figures 4C, D** **)**, as well as a significant increase in IL17 and IL6 concentration in the serum of mice. We also found that unlike CD4+ T cells, the CD8+ T cells were not significantly impacted by TCDD and FICZ. The CD8+ T cells have been shown previously to be down-regulated by TCDD (48, 49). This may be because the antigen that we used in this study, mBSA, is an exogenous antigen that activates primarily CD4+ Th1 and Th17 cells and not CD8+ T cells that are activated through the endogenous pathway of antigen processing and presentation (50). It should also be noted that antigen-activated T cells are more susceptible than naïve T cells to TCDD-mediated immunosuppression (51).

When we screened for IL22, we noted that TCDD caused a significant increase in IL22, whereas FICZ did not significantly alter IL22 expression. IL-22 is a member of the IL-10 family produced by a variety of immune cells, including Th1, Th17 cells, and Th22 cells (52). IL22 acts primarily on non-hematopoietic cells that express IL22 receptor and provides barrier immunity at the mucosal surfaces (53). TCDD has been shown previously to induce IL22 in human CD4+ T cells in an AhR-dependent manner (54). In another study, while TCDD induced IL22, FICZ was shown to suppress IL22 (55, 56). This contrasts with some studies showing that FICZ caused upregulation of IL22 (57). In the current study, we noted that FICZ did not alter the expression of IL22 in the MLN. Such discrepancies clearly suggest that the effect of AhR ligands on Th1, Th17, and Th22 cells may depend on a variety of factors, including binding affinity, nature of antigen, metabolism of the ligands, the target tissue, and the like.

In addition to the immunological changes seen in the MLNs during the DTH response, interestingly, we also saw significant changes in the colonic inflammation and microbiota induced by the DTH response. Currently, there are no published data indicating alterations in the gut microbiota during DTH response. It is likely that the mBSA antigen may travel to the MLN and the gut to induce an inflammatory response and consequently cause alterations in the gut microbiota or the antigen may directly cause alterations in the microbiota of the gut which may lead to a pro-inflammatory response. Additional studies are necessary to address this using cecal transplants of the microbiota.

Recently, several studies, including those from our laboratory, have shown that AhR plays a vital and important role in maintaining intestinal homeostasis, both when conditions are healthy and when not (58–62). In the current study, we noted that not only did the gut microbiota alter following DTH induction but also caused further changes in the microbiota in the administration of AhR ligands. The human gut microbiota consists primarily of two major bacterial phyla, Firmicutes and Bacteroidetes, which constitute more than 90% of the total community (63). It was thus interesting to note that DTH caused an increase in Bacteroidetes and a decrease in Firmicutes in the gut. Furthermore, TCDD caused a reversal of these effects while FICZ did not exert much change. These data are consistent with the ability of TCDD to suppress DTH response. While FICZ caused an increase in the DTH response, it did not correlate with an altered proportion of Firmicutes and Bacteroidetes, the reason for which was not clear but it can be speculated that the alterations in the microbiota had peaked with DTH induction. *Deferribacteres* is another gut microbiota with relatively less abundance which was found to be decreased during DTH response. TCDD was found to increase while FICZ did not cause major changes in these bacteria. AhR ligands have been well established to exert different levels of AhR activation, which is further influenced by AhR-induced metabolism. Thus, a single dose of TCDD triggers sustained AhR activation in the μg/kg range, whereas FICZ is rapidly metabolized and therefore causing transient AhR activation (35). This could lead to altered distribution or accumulation of FICZ vs TCDD in the gut which can, in turn, regulate the microbiota, independent of AhR. Clearly, additional studies are necessary to address this premise.

We also noted that DTH response altered levels of *Clostridium* spp and increased that of *Prevotella*. Furthermore, TCDD caused an increase in the abundance of *Clostridium* spp and decreased that of *Prevotella*, correlating with the ability of TCDD to suppress the DTH response, while FICZ did not have a major effect. Species of *Clostridium* cluster XIVa and IV are quite predominant in the gut, accounting for 10% to 40% of the total bacteria and act as key regulators of intestinal homeostasis (64, 65). Several studies have shown that increase in *Clostridium* spp, particularly clusters IV and XIVa, promoted Treg cell generation (64). Furthermore, colonization of mice by a defined mix of Clostridium strains facilitated an environment with high expression in TGF-β and affected Tregs number and functions in the colon (29). Furthermore, Atarashi et al using IL10 reporter mice demonstrated that Foxp3+ CTLA4^high^ Tregs induced by specific *Clostridium* species were adequately powerful to secrete IL10 to sustain both intestinal and systemic homeostasis (29). Our results are consistent with the above observations in that DTH induction led to a decrease in the abundance of *Clostridium* cluster XIV (*coccoides* group) and *Clostridium* cluster IV (*leptum* group) expression in the cecal flushes, and treatment with TCDD (mBSA+TCDD) caused marked increase in these *Clostridium* clusters as well as induction of Tregs in the MLNs and attenuation of DTH, while FICZ caused no marked changes in the *Clostridium* clusters, failed to induce Tregs and suppress DTH.

In recent years, several studies have shown a link between an increase in *Prevotella* bacteria and the disease development, as observed by the higher abundance of *Prevotella* spp in inflammatory disorders, such as rheumatoid arthritis, periodontitis, intestinal dysbiosis, and inflammation in HIV patients (8, 66–69). Interestingly, in the current study, we noted that DTH induction led to an increased abundance of *Prevotella copri* expression in the cecal flushes while treatment with TCDD caused a marked decline in *Prevotella copri* abundance while FICZ treatment caused a further increase in *Prevotella copri* abundance. These data were consistent with the observation that *Prevotella copri* may promote inflammation.

Changes in the gut microbiome often contribute to changes in bacterial metabolism, such as the production of SCFAs. SCFA, including acetic acid and butyric acid, are metabolic end products of undigested complex carbohydrate post bacterial fermentation throughout the colon (70). Our results showed that the level of acetic acid and butyric acid were significantly increased in mBSA+TCDD mice in comparison with mBSA+Veh mice. Zhang et al. showed that oral administration of sodium butyrate induced Tregs and inhibited IL17 production in plasma and colonic mucosa leading to amelioration of colonic colitis lesions in rats (71). Our laboratory has shown that administration of indole-3-carbinol (I3C), an AhR ligand into mice with colitis leads to attenuation of colitis which is associated with the induction of a subset of gram-positive bacteria known to produce butyrate, thereby causing increased production of butyrate (40). Additionally, butyrate administration also caused attenuation of colitis (40). In the current study, butyrate administration into mice with DTH was found to suppress the DTH response which was associated with an increased presence of Tregs and decreased levels of Th17 cells in the MLNs. Moreover, butyrate restored some of the beneficial bacteria such as *Clostridium* clusters while decreasing *Prevotella* that were deleterious.

Previous studies have suggested butyrate acts as a regulator of AhR-dependent genes and their functions, and acts as a histone deacetylase inhibitor (HDACi) (72). Also, butyrate has been shown to induce anti-inflammatory responses by inhibition of HDACs (46, 73). In this study, we also observed that there was a significant decrease in HDAC class I and II in mice with DTH and treated with TCDD when compared with mice with DTH treated with vehicle or FICZ. Moreover, butyrate was able to suppress HDACs class I and II in mice with DTH (**Figures 9C, D** **)**. Data taken together provides insights on how AhR ligands may have a potential role in modulating the cecal microbiome leading to induction of SCFA, HDAC inhibition, and amelioration of DTH in mice.

There are many reports demonstrating that TCDD and FICZ often exert opposing effects on T cell-mediated inflammation (10, 11). Our laboratory has shown that some dietary AhR ligands, such as I3C can also preferentially induce Tregs than Th17 (6, 12). The precise mechanisms responsible for the differential effects of AhR ligands to induce Tregs vs Th17 cells are not clear. Studies have suggested that this property may depend on the ligand, duration of AhR activation, the affinity of the ligand, epigenetic pathways induced, and the nature of microbiota [reviewed in (9)]. The current study supports the hypothesis that microbiota may play a role in the ability of AhR ligands to attenuate DTH response by promoting bacteria and butyrate production that induce Tregs. Additionally, our previous studies also demonstrated that TCDD may induce Tregs through regulation of miRNA-132 which targets HMGB1 (34).

In summary, the current study demonstrates that DTH induction in mice leads to significant alterations in the gut microbiota as well as associated proinflammatory T cell response in the MLNs and colon. Administration of TCDD leads to reversal of the alterations in the microbiota and attenuation of DTH which correlates with induction of Tregs, suppression of Th17 cells, and increased production of anti-inflammatory cytokines, such as TGF-β and IL-10. In contrast, FICZ enhances the DTH response, induces Th-17 cells but not Tregs, and fails to reverse the effect on microbiota induced during the DTH response. Together, our studies demonstrate that the ability of AhR ligands to suppress DTH response may depend on their capacity to reverse the alterations in the gut microbiota.

## Data Availability Statement

The datasets presented in this study can be found in online repositories. The names of the repository/repositories and accession number(s) can be found below: https://www.ncbi.nlm.nih.gov/sra, SRR14807275, SRR14807274, SRR14807273, SRR14807272, SRR14807271, SRR14807270, SRR14807280, SRR14807279, SRR14807278, SRR14807276, SRR14807277, SRR14807281.

## Ethics Statement

The animal study was reviewed and approved by the University of South Carolina Institutional Animal Care and Use Committee.

## Author Contributions

MN and PN designed the research studies, funding acquisition, resources, and supervision. OA performed all the experiments under the supervision of PN and MN. OA contributed to data acquisition, data analysis, writing, and original manuscript draft. OA, WN, MS, HA, and NS contributed to analyze data. NS, MN, PN, and PB contributed to writing, editing, and revisions. All authors contributed to the article and approved the submitted version.

## Funding

This study was supported in part by NIH grant nos. P01AT003961, P20GM103641, R01AT006888, R01ES030144, and R01AI123947 to PN and MN. OA, MS, and WN were supported by Iraqi HCED and MoHESR fellowships, respectively.

## Conflict of Interest

The authors declare that the research was conducted in the absence of any commercial or financial relationships that could be construed as a potential conflict of interest.

## Abbreviations

AhR, aryl hydrocarbon receptor; MLN, mesenteric lymph node; DTH, delayed-type hypersensitivity; ELISA, enzyme-linked immunosorbent assay; TCDD, 2,3,7,8-tetrachlorodibenzo-p-dioxin; FICZ, 6-formylindolo [3,2-b] carbazole; Foxp3, forkhead box P3; I3C, indole-3-carbinol; IL6, interleukin 6; IL17A, interleukin 17A; mBSA, methylated bovine serum albumin; RORγ, retinoic acid-related orphan receptor γ; RT-qPCR, reverse transcription-polymerase chain reaction; TGFβ, transforming growth factor-beta; Tregs, regulatory T cells; SCFA, short chain fatty acids; HDACs, histone deacetylases; IL10, interleukin 10; ISCs, intestinal stem cells; SFB, segmented filamentous bacterium; BUT, sodium butyrate; PBS, phosphate-buffered saline.

## References

[1] Justiz Vaillant AA Zulfiqar H Ramphul K. Delayed Hypersensitivity Reactions. StatPearls: StatPearls Publishing (2021). Available at: http://www.ncbi.nlm.nih.gov/books/NBK519023/. 30085565

[2] Dannenberg AM Jr. Roles of Cytotoxic Delayed-Type Hypersensitivity and Macrophage-Activating Cell-Mediated Immunity in the Pathogenesis of Tuberculosis. Immunobiology (1994) 191(4–5):461–73. 10.1016/S0171-2985(11)80452-3 7713560

[3] Dannenberg AM. Immune Mechanisms in the Pathogenesis of Pulmonary Tuberculosis. Rev Infect Dis (1989) 11(Supplement_2):S369–78. 10.1093/clinids/11.Supplement_2.S369 2496453

[4] Nakae S Komiyama Y Nambu A Sudo K Iwase M Homma I. Antigen-Specific T Cell Sensitization Is Impaired in IL-17-Deficient Mice, Causing Suppression of Allergic Cellular and Humoral Responses. Immunity (2002) 17(3):375–87. 10.1016/S1074-7613(02)00391-6 12354389

[5] Sakai M Yamashita K Takemoto N Ohshima Y Tsukimoto M Shinkai Y. Diesel Exhaust (DE) Aggravates Pathology of Delayed-Type Hypersensitivity (DTH) Induced by Methyl-Bovine Serum Albumin (Mbsa) in Mice. J Toxicological Sci (2009) 34(5):483–92. 10.2131/jts.34.483 19797856

[6] Singh NP Singh UP Rouse M Zhang J Chatterjee S Nagarkatti PS. Dietary Indoles Suppress Delayed-Type Hypersensitivity by Inducing a Switch From Proinflammatory Th17 Cells to Anti-Inflammatory Regulatory T Cells Through Regulation of MicroRNA. J Immunol (Baltimore Md.: 1950) (2016) 196(3):1108–22. 10.4049/jimmunol.1501727 PMC4724476 26712945

[7] Pregnane X Receptor as the “Sensor and Effector” in Regulating Epigenome. - Abstract - Europe Pmc. Available at: https://europepmc.org/article/med/25294580 (Accessed October 12, 2020).

[8] Gutiérrez-Vázquez C Quintana FJ. Regulation of the Immune Response by the Aryl Hydrocarbon Receptor. Immunity (2018) 48(1):19–33. 10.1016/j.immuni.2017.12.012 29343438 PMC5777317

[9] Prasad Singh N Nagarkatti M Nagarkatti P. From Suppressor T Cells to Regulatory T Cells: How the Journey That Began With the Discovery of the Toxic Effects of TCDD Led to Better Understanding of the Role of AhR in Immunoregulation. Int J Mol Sci (2020) 21(21)7849. 10.3390/ijms21217849 PMC7660163 33105907

[10] Quintana FJ Basso AS Iglesias AH Korn T Farez MF Bettelli E. Control of T Reg and T H 17 Cell Differentiation by the Aryl Hydrocarbon Receptor. Nature (2008) 453(7191):65–71. 10.1038/nature06880 18362915

[11] Kerkvliet NI Steppan LB Vorachek W Oda S Farrer D Wong CP. Activation of Aryl Hydrocarbon Receptor by TCDD Prevents Diabetes in NOD Mice and Increases Foxp3+ T Cells in Pancreatic Lymph Nodes. Immunotherapy (2009) 1(4):539–47. 10.2217/imt.09.24 PMC2823486 20174617

[12] Rouse M Singh NP Nagarkatti PS Nagarkatti M. Indoles Mitigate the Development of Experimental Autoimmune Encephalomyelitis by Induction of Reciprocal Differentiation of Regulatory T Cells and Th17 Cells. Br J Pharmacol (2013) 169(6):1305–21. 10.1111/bph.12205 PMC3831710 23586923

[13] Kawajiri K Fujii-Kuriyama Y. The Aryl Hydrocarbon Receptor: A Multifunctional Chemical Sensor for Host Defense and Homeostatic Maintenance. Exp Anim (2017) 66(2):75–89. 10.1538/expanim.16-0092 27980293 PMC5411294

[14] Ji J Qu H. Cross-Regulatory Circuit Between AHR and Microbiota. Curr Drug Metab (2019) 20(1):4–8. 10.2174/1389200219666180129151150 29380692

[15] Qin J Li R Raes J Arumugam M Burgdorf KS Manichanh C. A Human Gut Microbial Gene Catalogue Established by Metagenomic Sequencing. Nature (2010) 464(7285):59–65. 10.1038/nature08821 20203603 PMC3779803

[16] Kamada N Seo S-U Chen GY Núñez G. Role of the Gut Microbiota in Immunity and Inflammatory Disease. Nat Rev Immunol (2013) 13(5):321–35. 10.1038/nri3430 23618829

[17] Kim D Zeng MY Núñez G. The Interplay Between Host Immune Cells and Gut Microbiota in Chronic Inflammatory Diseases. Exp Mol Med (2017) 49(5):e339. 10.1038/emm.2017.24 28546562 PMC5454439

[18] Lee N Kim W-U. Microbiota in T-Cell Homeostasis and Inflammatory Diseases. Exp Mol Med (2017) 49(5):e340. 10.1038/emm.2017.36 28546563 PMC5454441

[19] Eisenstein EM Williams CB. The T(Reg)/Th17 Cell Balance: A New Paradigm for Autoimmunity. Pediatr Res (2009) 65(5 Pt 2):26R–31R. 10.1203/PDR.0b013e31819e76c7 19218879

[20] Atarashi K Tanoue T Oshima K Suda W Nagano Y Nishikawa H. T Reg Induction by a Rationally Selected Mixture of Clostridia Strains From the Human Microbiota. Nature (2013) 500(7461):232–6. 10.1038/nature12331 23842501

[21] Zhang H Liao X Sparks JB Luo XM. Dynamics of Gut Microbiota in Autoimmune Lupus. Appl Environ Microbiol (2014) 80(24):7551–60. 10.1128/AEM.02676-14 PMC4249226 25261516

[22] Zheng Y Dong C Yang J Jin Y Zheng W Zhou Q. Exosomal MicroRNA-155-5p From Pdlscs Regulated Th17/Treg Balance by Targeting Sirtuin-1 in Chronic Periodontitis. J Cell Physiol (2019) 234(11):20662–74. 10.1002/jcp.28671 31016751

[23] Mou H Wu S Zhao G Wang J. Changes of Th17/Treg Ratio in the Transition of Chronic Hepatitis B to Liver Cirrhosis and Correlations With Liver Function and Inflammation. Exp Ther Med (2019) 17(4):2963–8. 10.3892/etm.2019.7299 PMC6434237 30936966

[24] Al-Ghezi ZZ Singh N Mehrpouya-Bahrami P Busbee PB Nagarkatti M Nagarkatti PS. AhR Activation by TCDD (2,3,7,8-Tetrachlorodibenzo-P-Dioxin) Attenuates Pertussis Toxin-Induced Inflammatory Responses by Differential Regulation of Tregs and Th17 Cells Through Specific Targeting by MicroRNA. Front Microbiol (2019) 10:2349. 10.3389/fcimb.2019.02349 31681214 PMC6813193

[25] Shahid A Bharadwaj M. The Connection Between the Th17 Cell Related Cytokines and Cancer Stem Cells in Cancer: Novel Therapeutic Targets. Immunol Lett (2019) 213:9–20. 10.1016/j.imlet.2019.07.001 31278971

[26] Avalos-Navarro G Muñoz-Valle JF Daneri-Navarro A Quintero-Ramos A Franco-Topete RA Morán-Mendoza A. Circulating Soluble Levels of MIF in Women With Breast Cancer in the Molecular Subtypes: Relationship With Th17 Cytokine Profile. Clin Exp Med (2019) 19(3):385–91. 10.1007/s10238-019-00559-6 31102004

[27] Ivanov II Atarashi K Manel N Brodie EL Shima T Karaoz U. Induction of Intestinal Th17 Cells by Segmented Filamentous Bacteria. Cell (2009) 139(3):485–98. 10.1016/j.cell.2009.09.033 PMC2796826 19836068

[28] Wu H-J Ivanov II Darce J Hattori K Shima T Umesaki Y. Gut-Residing Segmented Filamentous Bacteria Drive Autoimmune Arthritis *Via* T Helper 17 Cells. Immunity (2010) 32(6):815–27. 10.1016/j.immuni.2010.06.001 PMC2904693 20620945

[29] Atarashi K Tanoue T Shima T Imaoka A Kuwahara T Momose Y. Induction of Colonic Regulatory T Cells by Indigenous Clostridium Species. Science (2011) 331(6015):337–41. 10.1126/science.1198469 PMC3969237 21205640

[30] den Besten G van Eunen K Groen AK Venema K Reijngoud D-J Bakker BM. The Role of Short-Chain Fatty Acids in the Interplay Between Diet, Gut Microbiota, and Host Energy Metabolism. J Lipid Res (2013) 54(9):2325–40. 10.1194/jlr.R036012 PMC3735932 23821742

[31] Ma X Zhou Z Zhang X Fan M Hong Y Feng Y. Sodium Butyrate Modulates Gut Microbiota and Immune Response in Colorectal Cancer Liver Metastatic Mice. Cell Biol Toxicol (2020) 36(5):509–15. 10.1007/s10565-020-09518-4 32172331

[32] Hegde VL Tomar S Jackson A Rao R Yang X Singh UP. Distinct MicroRNA Expression Profile and Targeted Biological Pathways in Functional Myeloid-Derived Suppressor Cells Induced by Δ9-Tetrahydrocannabinol In Vivo. J Biol Chem (2013) 288(52):36810–26. 10.1074/jbc.M113.503037 PMC3873541 24202177

[33] Sido JM Jackson AR Nagarkatti PS Nagarkatti M. Marijuana-Derived Δ-9-Tetrahydrocannabinol Suppresses Th1/Th17 Cell-Mediated Delayed-Type Hypersensitivity Through MicroRNA Regulation. J Mol Med (Berlin Germany) (2016) 94(9):1039–51. 10.1007/s00109-016-1404-5 PMC4992583 27038180

[34] Abdulla OA Neamah W Sultan M Chatterjee S Singh N Nagarkatti M. AhR Ligands Differentially Regulate MiRNA-132 Which Targets HMGB1 and to Control the Differentiation of Tregs and Th-17 Cells During Delayed-Type Hypersensitivity Response. Front Immunol (2021) 12:635903. 10.3389/fimmu.2021.635903 33679792 PMC7933657

[35] Ehrlich AK Pennington JM Bisson WH Kolluri SK Kerkvliet NI. Tcdd, FICZ, and Other High Affinity Ahr Ligands Dose-Dependently Determine the Fate of CD4+ T Cell Differentiation. Toxicological Sci (2018) 161(2):310–20. 10.1093/toxsci/kfx215 PMC5837604 29040756

[36] Singh NP Abbas IK Menard M Singh UP Zhang J Nagarkatti P. Exposure to Diethylstilbestrol During Pregnancy Modulates MicroRNA Expression Profile in Mothers and Fetuses Reflecting Oncogenic and Immunological Changes. Mol Pharmacol (2015) 87(5):842–54. 10.1124/mol.114.096743 PMC4407731 25753120

[37] Minett MS Eijkelkamp N Wood JN. Significant Determinants of Mouse Pain Behaviour. PloS One (2014) 9(8):e104458. 10.1371/journal.pone.0104458 25101983 PMC4125188

[38] Woolfe G Macdonald AD. The Evaluation of the Analgesic Action of Pethidine Hydrochloride (Demerol). J Pharmacol Exp Ther (1944) 80(3):300–7.

[39] Espejo EF Mir D. Structure of the Rat’s Behaviour in the Hot Plate Test. Behavioural Brain Research (1993) 56(2):171–6. 10.1016/0166-4328(93)90035-o 8240711

[40] Busbee PB Menzel L Alrafas HR Dopkins N Becker W Miranda K. Indole-3-Carbinol Prevents Colitis and Associated Microbial Dysbiosis in an IL-22–Dependent Manner. JCI Insight (2020) 5(1):e127551. 10.1172/jci.insight.127551 PMC7030851 31941837

[41] Nagalingam NA Kao JY Young VB. Microbial Ecology of the Murine Gut Associated With the Development of Dextran Sodium Sulfate-Induced Colitis. Inflamm Bowel Dis (2011) 17(4):917–26. 10.1002/ibd.21462 PMC3058753 21391286

[42] Lucke K Miehlke S Jacobs E Schuppler M. Prevalence of Bacteroides and Prevotella Spp. In *Ulcerative Colitis* . J Med Microbiol (2006) 55(Pt 5):617–24. 10.1099/jmm.0.46198-0 16585651

[43] Alrafas HR Busbee PB Nagarkatti M Nagarkatti PS. Resveratrol Modulates the Gut Microbiota to Prevent Murine Colitis Development Through Induction of Tregs and Suppression of Th17 Cells. J Leukocyte Biol (2019) 106(2):467–80. 10.1002/JLB.3A1218-476RR PMC6863607 30897248

[44] Akgun E Çaliskan C Celik H Ozutemiz A Tuncyurek M Aydin H. Effects of N-Acetylcysteine Treatment on Oxidative Stress in Acetic Acid-Induced Experimental Colitis in Rats. J Int Med Res (2005) 33(2):196–206. 10.1177/147323000503300207 15790131

[45] Weber N Liou D Dommer J MacMenamin P Quiñones M Misner I. Nephele: A Cloud Platform for Simplified, Standardized and Reproducible Microbiome Data Analysis. Bioinformatics (2018) 34(8):1411–3. 10.1093/bioinformatics/btx617 PMC5905584 29028892

[46] Segata N Izard J Waldron L Gevers D Miropolsky L Garrett WS. Metagenomic Biomarker Discovery and Explanation. Genome Biol (2011) 12(6):R60. 10.1186/gb-2011-12-6-r60 21702898 PMC3218848

[47] Alrafas HR Busbee PB Chitrala KN Nagarkatti M Nagarkatti P. Alterations in the Gut Microbiome and Suppression of Histone Deacetylases by Resveratrol Are Associated With Attenuation of Colonic Inflammation and Protection Against Colorectal Cancer. J Clin Med (2020) 9(6)1796. 10.3390/jcm9061796 PMC7355848 32526927

[48] Camacho IA Nagarkatti M Nagarkatti PS. Evidence for Induction of Apoptosis in T Cells From Murine Fetal Thymus Following Perinatal Exposure to 2,3,7,8-Tetrachlorodibenzo-P-Dioxin (TCCD). Toxicological Sci (2004) 78(1):96–106. 10.1093/toxsci/kfh048 14718643

[49] Nagarkatti PS Sweeney GD Gauldie J Clark DA. Sensitivity to Suppression of Cytotoxic T Cell Generation by 2,3,7,8-Tetrachlorodibenzo-P-Dioxin (TCDD) Is Dependent on the Ah Genotype of the Murine Host. Toxicol Appl Pharmacol (1984) 72(1):169–76. 10.1016/0041-008X(84)90261-8 6608808

[50] Roche PA Furuta K. The Ins and Outs of MHC Class II-Mediated Antigen Processing and Presentation. Nat Rev Immunol (2015) 15(4):203–16. 10.1038/nri3818 PMC6314495 25720354

[51] Singh NP Nagarkatti M Nagarkatti P. Primary Peripheral T Cells Become Susceptible to 2,3,7,8-Tetrachlorodibenzo-P-Dioxin-Mediated Apoptosis In Vitro Upon Activation and in the Presence of Dendritic Cells. Mol Pharmacol (2008) 73(6):1722–35. 10.1124/mol.107.043406 PMC2828294 18334599

[52] Parks OB Pociask DA Hodzic Z Kolls JK Good M. Interleukin-22 Signaling in the Regulation of Intestinal Health and Disease. Front Cell Dev Biol (2016) 3:85. 10.3389/fcell.2015.00085 26793707 PMC4710696

[53] Eken A Erdem S Haliloglu Y Okus FZ Cakir M Yetkin MF. Temporal Overexpression of IL-22 and Reg3γ Differentially Impacts the Severity of Experimental Autoimmune Encephalomyelitis. Immunology (2021). 10.1111/imm.13340 PMC8358722 33876425

[54] Brembilla NC Ramirez J-M Chicheportiche R Sorg O Saurat J-H Chizzolini C. In Vivo Dioxin Favors Interleukin-22 Production by Human Cd4+ T Cells in an Aryl Hydrocarbon Receptor (AhR)-Dependent Manner. PloS One (2011) 6(4)18741. 10.1371/journal.pone.0018741 PMC3078133 21525997

[55] Kiyomatsu-Oda M Uchi H Morino-Koga S Furue M. Protective Role of 6-Formylindolo[3,2-B]Carbazole (FICZ), an Endogenous Ligand for Arylhydrocarbon Receptor, in Chronic Mite-Induced Dermatitis. J Dermatol Sci (2018) 90(3)284–94. 10.1016/j.jdermsci.2018.02.014 29500077

[56] Um J-Y Kim H Kang Sy Son JH Chung BY Park CW. 2,3,7,8-Tetrachlorodibenzo-P-Dioxin Regulates the Expression of Aryl Hydrocarbon Receptor-Related Factors and Cytokines in Peripheral Blood Mononuclear Cells and CD4+ T Cells From Patients With Atopic Dermatitis and Psoriasis. Ann Dermatol (2020) 32(5):360–9. 10.5021/ad.2020.32.5.360 PMC7992582 33911769

[57] Monteleone I Rizzo I Sarra M Sica G Sileri P Biancone L. Aryl Hydrocarbon Receptor-Induced Signals Up-Regulate IL-22 Production and Inhibit Inflammation in the Gastrointestinal Tract. Gastroenterology (2011) 141(1)237–48. 10.1053/j.gastro.2011.04.007 21600206

[58] Pandiyan P Bhaskaran N Zou M Schneider E Jayaraman S Huehn J. Microbiome Dependent Regulation of Tregs and Th17 Cells in Mucosa. Front Immunol (2019) 10:426. 10.3389/fimmu.2019.00426 30906299 PMC6419713

[59] Omenetti S Pizarro TT. The Treg/Th17 Axis: A Dynamic Balance Regulated by the Gut Microbiome. Front Immunol (2015) 6:639. 10.3389/fimmu.2015.00639 26734006 PMC4681807

[60] Rannug A. How the AHR Became Important in Intestinal Homeostasis—A Diurnal FICZ/AHR/CYP1A1 Feedback Controls Both Immunity and Immunopathology. Int J Mol Sci (2020) 21(16)5681. 10.3390/ijms21165681 PMC7461111 32784381

[61] Safe S Jayaraman A Chapkin RS. Ah Receptor Ligands and Their Impacts on Gut Resilience: Structure–Activity Effects. Crit Rev Toxicol (2020) 50(6):463–73. 10.1080/10408444.2020.1773759 PMC7773274 32597352

[62] Neamah WH Busbee PB Alghetaa H Abdulla OA Nagarkatti M Nagarkatti P. AhR Activation Leads to Alterations in the Gut Microbiome With Consequent Effect on Induction of Myeloid Derived Suppressor Cells in a CXCR2-Dependent Manner. Int J Mol Sci (2020) 21(24):9613. 10.3390/ijms21249613 PMC7767008 33348596

[63] Hakansson A Molin G. Gut Microbiota and Inflammation. Nutrients (2011) 3(6):637–82. 10.3390/nu3060637 PMC3257638 22254115

[64] Nagano Y Itoh K Honda K. The Induction of Treg Cells by Gut-Indigenous Clostridium. Curr Opin Immunol (2012) 24(4):392–7. 10.1016/j.coi.2012.05.007 22673877

[65] Guo P Zhang K Ma X He P. Clostridium Species as Probiotics: Potentials and Challenges. J Anim Sci Biotechnol (2020) 11:24. 10.1186/s40104-019-0402-1 32099648 PMC7031906

[66] Alpizar-Rodriguez D Lesker TR Gronow A Gilbert B Raemy E Lamacchia C. Prevotella Copri in Individuals at Risk for Rheumatoid Arthritis. Ann Rheumatic Dis (2019) 78(5):590–3. 10.1136/annrheumdis-2018-214514 30760471

[67] Dillon S Lee E Kotter C Austin G Gianella S Siewe B. Gut Dendritic Cell Activation Links an Altered Colonic Microbiome to Mucosal and Systemic T Cell Activation in Untreated HIV-1 Infection. Mucosal Immunol (2016) 9(1):24–37. 10.1038/mi.2015.33 25921339 PMC4626441

[68] Pedersen HK Gudmundsdottir V Nielsen HB Hyotylainen T Nielsen T Jensen BAH. Human Gut Microbes Impact Host Serum Metabolome and Insulin Sensitivity. Nature (2016) 535(7612):376–81. 10.1038/nature18646 27409811

[69] Scher JU Sczesnak A Longman RS Segata N Ubeda C Bielski C. Expansion of Intestinal Prevotella Copri Correlates With Enhanced Susceptibility to Arthritis. eLife (2013) 2:e01202. 10.7554/eLife.01202 24192039 PMC3816614

[70] Cummings JH Pomare EW Branch WJ Naylor CP Macfarlane GT. Short Chain Fatty Acids in Human Large Intestine, Portal, Hepatic and Venous Blood. Gut (1987) 28(10):1221–7. 10.1136/gut.28.10.1221 PMC1433442 3678950

[71] Zhang M Zhou Q Dorfman RG Huang X Fan T Zhang H. Butyrate Inhibits Interleukin-17 and Generates Tregs to Ameliorate Colorectal Colitis in Rats. BMC Gastroenterol (2016) 16:84. 10.1186/s12876-016-0500-x 27473867 PMC4967301

[72] Jin U-H Cheng Y Park H Davidson LA Callaway ES Chapkin RS. Short Chain Fatty Acids Enhance Aryl Hydrocarbon (Ah) Responsiveness in Mouse Colonocytes and Caco-2 Human Colon Cancer Cells. Sci Rep (2017) 7:10163. 10.1038/s41598-017-10824-x 28860561 PMC5579248

[73] Patnala R Arumugam TV Gupta N Dheen ST. Hdac Inhibitor Sodium Butyrate-Mediated Epigenetic Regulation Enhances Neuroprotective Function of Microglia During Ischemic Stroke. Mol Neurobiol (2017) 54(8):6391–411. 10.1007/s12035-016-0149-z 27722928

